# No evidence for accumulation of deleterious mutations and fitness degradation in clonal fish hybrids: Abandoning sex without regrets

**DOI:** 10.1111/mec.15539

**Published:** 2020-08-04

**Authors:** Jan Kočí, Jan Röslein, Jan Pačes, Jan Kotusz, Karel Halačka, Ján Koščo, Jakub Fedorčák, Nataliia Iakovenko, Karel Janko

**Affiliations:** ^1^ Department of Biology and Ecology University of Ostrava Ostrava Czechia; ^2^ Institute of Animal Physiology and Genetics Czech Academy of Science Liběchov Czechia; ^3^ Institute of Molecular Genetics Czech Academy of Science Prague Czechia; ^4^ Museum of Natural History University of Wrocław Wrocław Poland; ^5^ Institute of Vertebrate Biology Czech Academy of Science Brno Czechia; ^6^ Department of Ecology University of Prešov Prešov Slovakia

**Keywords:** asexuality, clonal decay, exome capture, fitness, Muller’s ratchet, mutation load

## Abstract

Despite its inherent costs, sexual reproduction is ubiquitous in nature, and the mechanisms to protect it from a competitive displacement by asexuality remain unclear. Popular mutation‐based explanations, like the Muller's ratchet and the Kondrashov's hatchet, assume that purifying selection may not halt the accumulation of deleterious mutations in the nonrecombining genomes, ultimately leading to their degeneration. However, empirical evidence is scarce and it remains particularly unclear whether mutational degradation proceeds fast enough to ensure the decay of clonal organisms and to prevent them from outcompeting their sexual counterparts. To test this hypothesis, we jointly analysed the exome sequences and the fitness‐related phenotypic traits of the sexually reproducing fish species and their clonal hybrids, whose evolutionary ages ranged from F1 generations to 300 ky. As expected, mutations tended to accumulate in the clonal genomes in a time‐dependent manner. However, contrary to the predictions, we found no trend towards increased nonsynonymity of mutations acquired by clones, nor higher radicality of their amino acid substitutions. Moreover, there was no evidence for fitness degeneration in the old clones compared with that in the younger ones. In summary, although an efficacy of purifying selection may still be reduced in the asexual genomes, our data indicate that its efficiency is not drastically decreased. Even the oldest investigated clone was found to be too young to suffer fitness consequences from a mutation accumulation. This suggests that mechanisms other than mutation accumulation may be needed to explain the competitive advantage of sex in the short term.

## 1 INTRODUCTION

The evolutionary success of sex has been one of the greatest puzzles evolutionary biologists have pondered for over a century (Weismann, 1889). Although, theoretically, sexually reproducing organisms should be outcompeted by the asexual organisms due to inherent costs of sex, they remain predominant in the world of eukaryotes. Numerous hypotheses have been proposed to explain this paradox (see Shcherbakov, 2010, for one recent hypothesis, reviewed in, e.g., Hartfield & Keightley, 2012). Some are linked to inherent ecological properties of reproductive modes, such as the penalization of asexuals for their inability to rapidly generate novel variants, thus increasing their intraspecific competition (Doncaster, Pound, & Cox, 2000; McDonald, Rice, & Desai, 2016; Peck, Yearsley, & Waxman, 1998) or vulnerability to pathogens (Hamilton, 1980). Another popular class of theories emphasizes an increased extinction risk of the clones (ultimately decreasing their diversification), or the gradual deterioration of their genomes (hereafter referred to as mechanisms of “clonal decay”). These are based on the hypothesis that every asexual lineage will acquire harmful mutations, and selection will not be efficient enough to maintain the fittest genome (Kondrashov, 1982) because individual mutations may not escape their genomic background due to a whole‐genome linkage, leading to accumulation of mutations in a stochastic (Muller's ratchet) or a deterministic (Kondrashov's hatchet) manner (Kondrashov, 1993). Consistent with this hypothesis, most asexual lineages appear to be evolutionarily short‐lived, and occupy terminal positions on the tree of life (reviewed in Janko, Drozd, Flegr, & Pannell, 2008; Schwander & Crespi, 2009). Several genomic studies indeed have found evidence of higher rates of accumulation of nonsynonymous mutations in asexuals (e.g., Henry, Schwander, & Crespi, 2012; Hollister et al., 2015; Howe & Denver, 2008; Johnson & Howard, 2007; Neiman, Hehman, Miller, Logsdon, & Taylor, 2010; Paland & Lynch, 2006).

Despite broad consensus that accumulation of mutation should ultimately lead to the demise of asexual lineages, it remains unclear whether the process proceeds fast enough to exterminate the asexuals before they become competitive threats to the sexual populations. Some argue that higher rates of mutation accumulation can considerably harm the clonal populations even over a short period of time (e.g., Neiman et al., 2010), especially in synergy with other mechanisms (Pound, Cox, & Doncaster, 2004; West, Lively, & Read, 1999). However, a non‐negligible number of studies have failed to confirm higher loads of mutations in asexual organisms (e.g., Allen, Light, Perotti, Braig, & Reed, 2009; Brandt et al., 2017; Naito & Pawlowska, 2016; Pellino et al., 2013; Warren et al., 2018), and several studies have also demonstrated comparable or higher net diversification rates in asexual clades than in the sexual ones, suggesting these lineages may not inherently suffer from a decreased diversification, an increased extinction or both (Fontaneto, Tang, Obertegger, Leasi, & Barraclough, 2012; Johnson, FitzJohn, Smith, Rausher, & Otto, 2011; Liu et al., 2012).

Such contradictions do not necessarily invalidate detrimental effects of mutation accumulation as a factor, but they indicate that the full explanation is more complex. For example, asexuals may have some mechanisms that temporarily delay the process of clonal decay, such as “minimal sex,” ameiotic recombination, gene conversions, increasing ploidy with consequent genome refreshment and masking mutations with heterozygous states (e.g., Flot et al., 2013; Halligan & Keightley, 2003; Loewe & Lamatsch, 2008; Sémon & Wolfe, 2007). It is also possible that reduction in efficacy of selection stemming from asexuality is not drastic enough to universally cause the expected trends in mutation accumulation patterns (Allen et al., 2009). It thus remains a major challenge in evolutionary biology to identify the short‐term‐acting mechanisms that protect the sexuals from competitive displacement by the asexuals.

The so‐called sexual–asexual complexes, that is taxa in which related sexual and asexual forms coexist, play prominent roles in studies of the persistence of sex since both types of organisms enter the same evolutionary arena for mutation, selection and drift (Birky & Barraclough, 2009; Janko, Drozd, & Eisner, 2011). In some complexes, asexual lineages may emerge from the sexual populations very dynamically (Janko et al., 2012), which poses an additional question regarding the role of mutation accumulation process in saving sex: even if individual clonal lineages become debilitated in a relatively short term, whole asexual populations, composed of multiple frequently emerging clones, may escape extinction and pose a serious short‐term or long‐term threat to the sexual counterparts (Paland, Colbourne, & Lynch, 2005). Interestingly, Janko et al. (2008) showed that continuous influx of new clones into an asexual population causes inevitable replacement of the old clones by drift without invoking an age‐dependent decay of the clonal lineages. This implies that ages attained by the individual clonal lineages may not correspond to their theoretical maxima determined by an age‐dependent decay, but rather by the rate of influx of the new clones into an asexual community of a finite size. This drift‐like clonal turnover process proved sufficient to explain some phylogenetic data (Janko, 2014), suggesting that in some cases clones may vanish from a population before mutation accumulation or other such processes compromise their fitness.

Even less is known about the direct impact of mutation accumulation on clone fitness. Indeed, if increased mutation accumulation in asexuals is relevant to the maintenance of sex, it should have phenotypic consequences when relatively old asexual lineages “… suffer in comparison to younger asexual lineages (and sexuals) with regard to important traits such as mitochondrial function and the rate of offspring production and population growth” (Neiman et al., 2010). Unfortunately, studies investigating fitness‐related traits are scarce, and their results are contradictory; age‐dependent fitness deterioration has been demonstrated in some lineages of viruses, bacteria and yeasts (Andersson & Hughes, 1996; Chao, 1990; Goddard, Godfray, & Burt, 2005), but not in water frogs with genomes transmitted clonally for over 25 ky (Guex, Hotz, & Semlitsch, 2002).

In this study, we focused on the *Cobitis taenia* hybrid complex of the European bottom‐dwelling loaches, and examined whether the genomes of sexual, young asexual and old asexual lineages differed in accumulation of nonsynonymous mutations, and whether the old and the young clones differed in traits that impacted their fitness.

**Figure 1:**
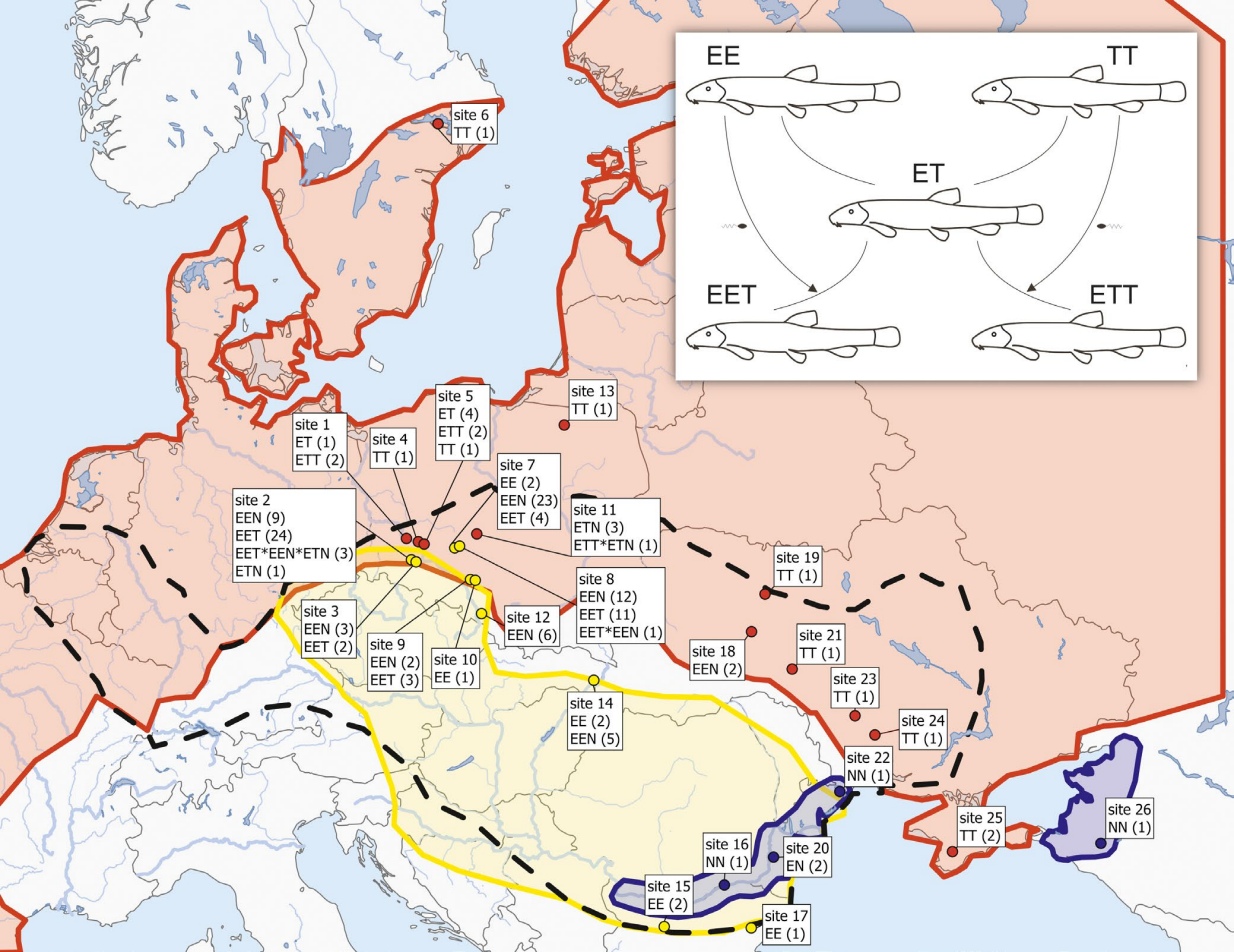
Sampling sites of *Cobitis* study materials. E, T, N—genotypes of *C. elongatoides*, *C. taenia* and *C. tanaitica*, respectively; number of specimens in parentheses. Red area = range of *C. taenia*; yellow area = range of *C. elongatoides*; blue area = range of *C. tanaitica*. Dashed line delimitates range of the oldest asexual lineage, the “hybrid clade I” [Colour figure can be viewed at wileyonlinelibrary.com]

Our model taxon consists of several parapatrically distributed sexual species that inhabit Europe and coexist over much of the continent with their hybrids that regularly establish successful, persistent clonal lineages with a gynogenetic reproductive mode (i.e., females lay unreduced eggs that require activation by sperm from sexual males to trigger development). Production of clonal eggs is achieved via “premeiotic endomitosis” when oogonial chromosomes are duplicated, and subsequent meiotic divisions do not yield any variability since crossovers occur among identical copies of chromosomes (Dedukh et al., 2019;Juchno, Arai, Boroń, & Kujawa, 2016). Phylogenetic and crossing experiments (Choleva et al., 2012; Janko, Bohlen, et al., 2007; Janko, Culling, Rab, & Kotlik, 2005; Janko et al., 2012; Janko, Vasil’ev, et al., 2005) showed that range shifts related to the Pleistocene climatic oscillations have repeatedly placed the *Cobitis* parental species into a secondary reproductive contact, provoking the dynamic formation of hybrid clones. Their diversity is created in a two‐step process (Figure 1 insert). First, diploid clonal F1 females form through crosses of the parental species *C. elongatoides* with either *C. taenia* or *C. tanaitica* (the hybrid males are sterile; Choleva et al., 2012; Janko et al., 2018); for simplicity, the haploid genomes of those species have been hereafter denoted as E, T and N, respectively, and hybrid forms with particular combinations of the parental genome (so‐called genomotypes) have been denoted by these letters; for example, ETT indicates a triploid hybrid with one elongatoides and two taenia genomes. In the second step, mating with sexual males may occasionally result in the incorporation of sperm genomes leading to the formation of secondary triploid clones. Tetraploids are occasionally produced in an analogous way, but generally die before maturity (Janko, Bohlen, et al., 2007; Juchno et al., 2014). Contemporary *Cobitis* populations are composed of a mixture of clones of quite different evolutionary ages. Some clones are very recent. Other clones originated during the early Holocene with establishment of the secondary contact zones between the parental species. Molecular dating using mtDNA data indicates that the origin of the oldest known lineage dates to ~0.3 Mya (Janko, Culling, et al., 2005; Majtánová et al., 2016). This lineage, referred to as the “hybrid clade I,” originated in the Ponto‐Caspian region as a diploid *C. elongatoides–tanaitica* (EN) hybrid, and during its subsequent expansion, it has incorporated additional genomes on multiple occasions, resulting in a monophyletic clonal assemblage of the EN, the EEN, the ENN and the ETN clonal genomotypes. Owing to a lack of evidence on the observation of asex → sex transitions, the emergence of asexuality in the *Cobitis* appears to be a unidirectional process that has been more or less continuously occurring for at least hundreds of thousands of years, which should be enough to observe the putative effects of clonal decay (Loewe & Lamatsch, 2008). Mechanisms ensuring such long‐term coexistence with sexual forms have not been properly understood, although Kotusz et al. (2014) and Bartoš et al. (2019) documented niche segregation between the *Cobitis* forms, suggesting some role of ecological factors.

Dynamic emergence of asexuality, the presence of diploid and polyploid clones, and a relatively well‐resolved evolutionary history and ecology all make the *Cobitis* an attractive model for investigation of genomic and phenotypic consequences of asexual reproduction, allowing, among other objectives, the disentanglement of polyploidy effects from asexuality and/or hybridization. Here, we simultaneously analysed the genetic variability in nuclear and mitochondrial genes, together with fitness data, to test predictions of mutation accumulation hypotheses. Specifically, we investigated whether asexual genomes carried traces of reduced efficacy of purifying selection, which may be manifested by *d*_N_/*d*_S_ ratios closer to 1 along the “asexual” branches of the phylogenetic trees (Wertheim et al., 2015), by generally higher rates of accumulation of the nonsynonymous mutations or by more radical amino acid substitutions in clonal genomes (Pellino et al., 2013; Sharbrough, Luse, Boore, Logsdon, & Neiman, 2018). We further compared mutation frequency spectra to test whether the spread of novel nonsynonymous variants differed between the sexual and the asexual lineages (Hollister et al., 2015). Finally, we extended our analyses to the phenotypic data from additional individuals and tested whether traits related to the body condition and fecundity tended to decay in the ageing clones.

## 2 MATERIALS AND METHODS

Origins and numbers of analysed specimens are listed in Figure 1 and Tables 1 and 2. The taxonomical identities of all fish used in this study were determined with a set of previously published and validated species diagnostic markers including allozyme and microsatellite loci and flow cytometric determination of ploidy (Janko, Flajšhans, et al., 2007; Janko et al., 2012).

**Table 1:** Overview of samples used in genomic part of the study

Genomotype	No. of individuals	Category	Clone ID
EE	8	Sexual	NA
NN	4	Sexual	NA
TT	10	Sexual	NA
LL	1	Outgroup	NA
EEN	10	Old clone	9 (10)
EET	5	Young clone	1 (3), 16 (2)
ET	7	F1, young clone	F1 (2), 2d (1), 10d (2), 12d (1), 67 (1)
EN	2	Old clone	NA
ETT	4	Young clone	53 (1), 61 (1), 65 (1)

Note

Upper‐case letters represent a haploid genome of these species: E = *C. elongatoides*; L = *C. paludica*; N = *C. tanaitica*; T = *C. taenia*; for example, EET stands for triploid with two genomes of *C. elongatoides* and one of *C. taenia*. Categories were used to divide data sets for use in linear models (LME/GLM/GLME), where sexuals were also used for interspecific differences between pairs of species. F1 denotes F1 laboratory hybrids. Clone ID denotes the membership of given individual to particular multilocus lineage inferred from SNP data, which represent independent clonal lineage, with number of individuals per lineage in parentheses.

**Table 2:** Locality information and number of *Cobitis* specimens used in the study concerning fitness proxy traits

River	Site		No. of spec. old clone	No. of spec. young clones	Collection dates
	Latitude	Longitude	(spring/fall)	(spring/fall)	
Skora	51°17′	16°05′	(3/5)	(4/20)	21.04.2005, 12.09.2013
Złotnica	51°33′	17°41′	0/12	0/12	11.09.2013
Polska Woda	51°31′	17°30′	0/23	0/4	12.09.2013
Swędrnia^a^	51°49′	18°15′	0	0/4	11.09.2013
Budkowiczanka	50°52′	18°02′	(1/0)	(2/0)	22.04.2005
Wierzbiak	51°14′	16°14′	(2/0)	(2/0)	21.04.2005
Sumida	50°09′	18°24′	(6/0)	0	22.04.2005
Total			12/40	10/40	

^a^ The sample where the parental species was *C. taenia*, on the remaining sites, parental was *C. elongatoides*.

### 2.1 Genomic data

Exome data were acquired using the exome capture method described in Janko et al. (2018) and briefly below. Using probes for targeted enrichment of gDNA loci, we collected sequence data from nuclear exomic and mtDNA segments of 22 females belonging to three hybridizing sexual species, 26 hybrids of different ploidy, and the genome composition and one specimen of *Cobitis paludica* as an outgroup (Table 1; Figure 1). Samples of parental species represent all major phylogroups, sensu Janko, Culling, et al. (2005), to capture maximum coverage of intraspecific variability in our data. Hybrid specimens include two or more individuals from several independent clonal lineages characterized previously by Janko et al. (2012), to compare genetic variability within and between clonal lineages.

#### 2.1.1 Sequencing and SNP calling

Isolated gDNA was sheared with Bioruptor, tagged by indices, pooled and hybridized to custom‐designed sequence‐capture probes. Captured fragments were sequenced on an Illumina NextSeq in 75 bp paired‐end (PE) mode. Fastq files were trimmed by the fqtrim tool (Pertea, 2015) with minimum read lengths of 20 bp, and 3′ end trimming when average base quality within a sliding window drops below 15, which is a commonly recommended threshold (e.g., Suren et al., 2016).

To further process the reads, we used a published *Cobitis* reference transcriptome (GGJF00000000; Janko et al., 2018), from which Janko et al. (2018) discarded potentially paralogous contigs, as described in Gayral et al. (2013). In short, we considered as potentially paralogous contigs those that possessed spurious heterozygosity patterns when the same heterozygotic positions occurred across distantly related species (including the outgroup). Such patterns are unlikely to originate from meaningful biological processes, and probably result from mapping of paralogous reads onto one contig present in the reference. Loci with such properties were removed from the reference. For the purposes of this study, we annotated the reference transcriptome with blast2go software (Conesa et al., 2005). Contigs were aligned against the nr database (18.11.2015) by blastx 2.2.31. Hits with *e*‐values <0.0001 were accepted as significant. Otherwise, default settings were used. Subsequently, we identified the longest open reading frame (ORF) within each annotated contig using the getorf tool from embassy package (version 6.6.0.0).

We aligned reads to this reference with bwa MEM (Li & Durbin, 2009), followed by picard tools version 1.140 to mark duplicates (Broad Institute, http://broadinstitute.github.io/picard). Individuals’ variants were called with the gatk version 3.4 HaplotypeCaller tool, and all individuals were jointly genotyped using the genotypegvcfs tool (Van der Auwera et al., 2013; DePristo et al., 2011; McKenna et al., 2010). Variant recalibration was based on a previously used database of species diagnostic positions (Janko et al., 2018) representing a learning set for the variant quality score recalibration tool VariantRecalibrator, and all variants were then filtered with the applyrecalibration tool. All resulting high‐confidence SNPs with coverage ≥5 were transferred to a database using custom SQL/python scripts.

Four samples represent an experimental family including two *C. elongatoides–taenia* F1 hybrid individuals and both their parents, which allowed us to experimentally verify the efficiency of sequencing and SNP calling by comparing F1 hybrids with their parents. We also compared our NGS data with Sanger sequences of several loci obtained by Choleva et al. (2014) to assess the accuracy of our method.

#### 2.1.2 Tests of relaxed selection in mitochondrial genomes

Because haploid mtDNA data are not biased by the problem of phasing, we first analysed mitochondrial genomes by mapping reads to published *C. elongatoides* mitochondrion (Accession no. NC_023947.1) and applying the relax software (Wertheim et al., 2015) to individual haplotypes to test the hypothesis that selection is relaxed along asexual branches, resulting in *d*_N_/*d*_S_ ratios closer to 1 than along sexual branches. Since the software requires phased sequences representing single ORF with no heterozygous sites or stop codons, we discarded all noncoding sequences (D‐loop and tRNAs), and the reverse‐oriented ND6 gene from our mtDNA reference. We also masked several observed heterozygous positions and stop codons with “N” using the hyphy package (Pond, Frost, & Muse, 2005), since these may represent sequencing errors or mutated premature termination codons. In case of overlapping ORFs in several mitochondrial genes, we split the sequence at the start codon of the second ORF, and padded the 3′ end of the first ORF with “N” to keep the correct reading frame of the whole alignment.

**Figure 2:**
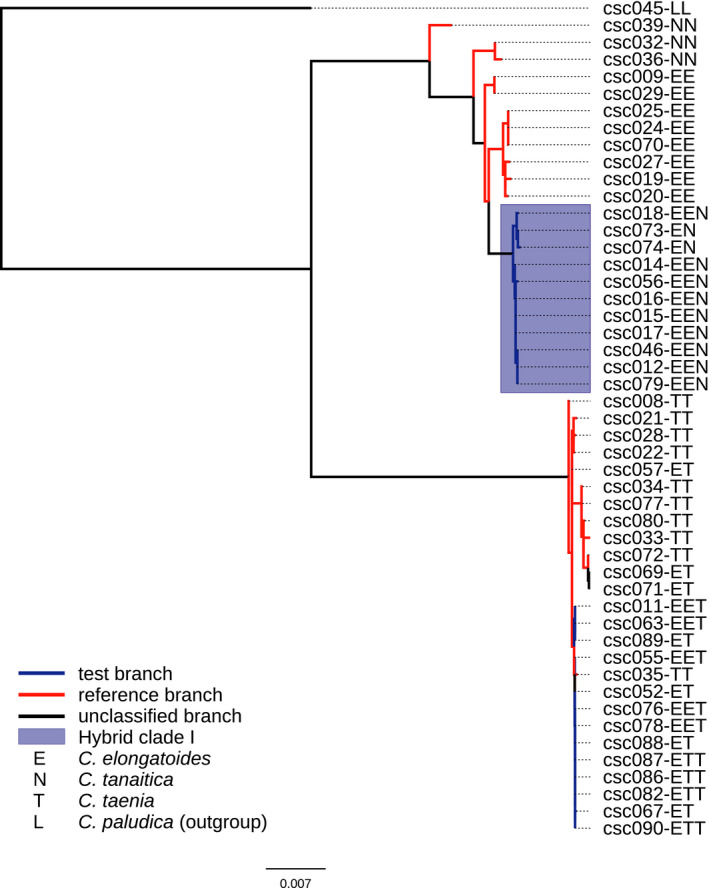
Maximum likelihood‐based reconstruction of phylogenetic relationships of mtDNA haplotypes as input for relax analysis. Asexual branches were tested (blue) against intraspecific sexual branches, which served as reference branches (red) in the relax model. Blue box represents lineages of the oldest asexual lineage, the “Hybrid clade I.” Letter codes after each sample indicate its genomotype [Colour figure can be viewed at wileyonlinelibrary.com]

The final alignment of coding mtDNA was uploaded to the phyml server (version 3.0 build 20120412; Guindon et al., 2010), and phylogenetic trees were computed with default settings. A substitution model was selected based on AIC using Smart Model Selection (sms 1.8.1.; Lefort, Longueville, & Gascuel, 2017; Figure 2, Table S1). We then used both the alignment and Newick tree file in hyphy 3.8 to perform relaxed selection analysis in relax with the assumption of strict sex → asex transitions, which is justified based on known pathways of asexual hybridization in *Cobitis* (Choleva et al., 2012). The software requires two types of branches to be specified in each tree. As test branches, we selected those leading exclusively to asexual individuals (i.e., asexual branches). As reference branches, we considered the intraspecific tree branches within all three sexual species (i.e., sexual branches; Figure 2). We left the interspecific branches unclassified, since sequence evolution at the interspecific level is likely to bear traces of stronger negative selection, as many mildly deleterious mutations segregating within species would not become fixed between species. Choleva et al. (2014) showed that *C. tanaitica* is fixed for introgressed elongatoides‐derived mitochondrion and appears paraphyletic to *C. elongatoides*, potentially indicating repeated introgression events in the past. Therefore, we also left one internal branch within *C. tanaitica* unclassified, as we could not determine whether it represents an intra‐ or interspecific branch (see Figure 2). We also left unclassified the branches leading to laboratory progeny used in F1 crosses, as they represent non‐natural experimental strains with unknown reproductive modes in the F1 generation.

We compared the fit with data of the two models allowing site‐specific *d*_N_/*d*_S_ ratios, that is the null model assuming the same ratios for asexual (test) and sexual (reference) branches of the ML phylogenetic tree (see Figure 2), and the alternative model assuming that *d*_N_/*d*_S_ ratios differ between types of branches.

#### 2.1.3 Tests of reduced efficacy of selection in nuclear DNA

While mitochondrial data allowed the application of sophisticated methods (i.e., relax), we were also interested in trends in nuclear DNA, which is highly heterozygous in hybrids and thus had to be analysed differently. Specifically, we inspected whether ratios of synonymous and nonsynonymous mutations differ between sexual and asexual populations by comparing the frequency spectra in each population, and testing whether radicality of nonsynonymous substitutions differs among genomotypes. Finally, as a more formal test of selection, we compared per‐gene distributions of *d*_N_/*d*_S_ ratios among data sets. Details are provided below.

##### Types of SNPs, N/S ratios, site frequency spectra and radicality of amino acid substitutions

All nDNA analyses are based on ORFs that represent annotated protein‐coding sequences with assigned GO terms. For methodological simplicity (see e.g., Burgarella et al., 2015), we constrained all downstream analyses to biallelic SNP positions, that is those with a maximum of two alternative alleles observed across all investigated specimens (note that less than ~0.001 of positions contained more than two variants, similar to observations by Ament‐Velásquez et al., 2016). We further masked with “N” all triplets containing more than one polymorphic site since we could not be sure about their amino acid translation without knowing their phase. Also, to enter the SNP database, each site had to be successfully sequenced in at least 80% of individuals. After filtering, each SNP was categorized into one of the three following categories. First, we identified sites segregating within any of the three parental species (i.e., intraspecific sexual polymorphisms). Second, we scored SNP variants that were monomorphic within each species but differed between some pairs of species (i.e., fixed interspecific sexual polymorphisms). Finally, we noted the so‐called private asexual SNPs, where all parental sexual species appeared invariant, but asexual hybrids carried apomorphic alleles. We assumed that such substitutions generally accumulated after the origin of hybrid clonal lineages (see below).

To determine synonymity of any mutation, we translated nucleotide sequences into amino acids (including premature stop codons) using r package seqinr and compared numbers of synonymous and nonsynonymous mutations among all SNP types (intraspecific and interspecific sexual, and private asexual). Although some tools exist for direct comparisons of selection efficiency and distributions of mutation fitness effects between populations (Eyre‐Walker & Keightley, 2009) and have even been applied to sexual–asexual comparisons (e.g., Hollister et al., 2015), some of their assumptions (segregation and recombination) make their usage for asexuals problematic; hence, we did not apply them. Instead, we also estimated the frequency of each SNP in sexual populations and within each clonal lineage, and tested whether patterns differ between synonymous and nonsynonymous substitutions. For this analysis, we assumed that the ancestral state of each intraspecific or private asexual SNP corresponded to the variant present in the sister species, while the other allele was assumed to be mutated.

We also tested whether radicality of acquired nonsynonymous mutations could be higher in asexuals because lack of segregation in asexuals should reduce the efficacy of selection against mutations with strong effects, especially when the clone possesses another functional copy of a given gene (Hollister et al., 2015; Sharbrough et al., 2018). We performed two tests of this hypothesis. We first calculated for all categories of SNPs the proportions of polymorphic sites carrying premature stop codons, which likely represent strong‐effect mutations. Second, we scored the “radicality” of nonsynonymous mutations with BLOSUM90 and PAM100 substitution matrices, and compared score distributions among sexual and asexual data sets. We have chosen these matrices due to low numbers of mutation per gene, which may leave some amino acid substitutions unobserved.

##### *d*_N_/*d*_S_ ratio

The *d*_N_/*d*_S_ ratio, that is the ratio of the number of nonsynonymous substitutions per nonsynonymous site (*d*_N_) to the number of synonymous substitutions per synonymous site (*d*_S_), is a common technique used to detect selection in pairs of protein‐coding sequences. Comparison of *d*_N_/*d*_S_ ratios between pairs of sexual and asexual lineages is a suitable tool to address the efficiency of selection with the prediction that asexuals should possess higher *d*_N_/*d*_S_ values due to reduced efficacy of purifying selection (e.g., Pellino et al., 2013). However, due to the hybrid origin of asexuals, their *d*_N_/*d*_S_ values are not directly comparable to those from sexual species. Many of asexuals’ polymorphisms are inherited from substitutions fixed between parental species brought together by hybridization, and we know their *d*_N_/*d*_S_ are lower than those of intraspecific polymorphisms segregating within parental species. Thus, we also calculated *d*_N_/*d*_S_ ratios for all possible *elongatoides–taenia* and *elongatoides–tanaitica* interspecific pairs, which served as null distributions to be compared with real asexual hybrids. This approach is based on the rationale that *d*_N_/*d*_S_ ratios of simulated hybrids provide variability that would be expected in hybrids without the effects of any process operating on asexual genomes.

For comparisons of *d*_N_/*d*_S_ distributions within and between species and genomotypes, we selected ORFs with at least 50 intact codons fully resolved in all individuals, to maintain representative sequence lengths. We calculated *d*_N_/*d*_S_ ratios for all pairs of sequences using the Nei–Gojobori evolutionary pathway method and the Jukes–Cantor model as implemented in the bioperl module Bio::Align::DNAStatistics (http://www.bioperl.org; Stajich et al., 2002). Since some sequence pairs differed in synonymous or nonsynonymous mutations, but not both, which would introduce zeros into numerators or denominators of *d*_N_/*d*_S_ ratios, we added a small constant to each estimate (0.01; Pellino et al., 2013).

### 2.2 Estimating fitness effects in old versus recent clones

Inspection of SNPs, albeit very instructive, may only provide an indirect proxy for inferences about fitness decay in clones. The next logical step, albeit rarely undertaken, is to test for age‐dependent fitness deterioration of clones. To make such a comparison, we examined additional samples from 7 sites in the Central European hybrid zone, where asexuals belonging to recent and ancient lineages coexist. All specimens used for phenotypic analyses were genetically assessed by standard markers to determine their genomotype (Janko, Bohlen, et al., 2007).

Because diploid and triploid *Cobitis* hybrids differ in many traits (Bobyrev, Burmensky, Vasilev, Kriksunov, & Lebedeva, 2003; Juchno & Boron, 2010; Kotusz, 2008; Maciak et al., 2011), we restricted analyses to triploid females, and investigated traits related to body condition, growth and fecundity, predicting more prominent fitness decay in older clones compared with recent ones (Neiman et al., 2010). For this part of the study, we captured and analysed 48 EEN females belonging to the ancient (hybrid clade I) and 52 females belonging to recent clonal lineages (EET and ETT genomotypes; Table 2; Figure 1). To address seasonal variation, we performed sampling before and after the reproductive season (April and September, respectively). We caught the fish by electrofishing, sacrificed them by overdose of 2‐di‐phenoxyethanol and fixed them in 4% formaldehyde after removing a piece of fin tissue for genotyping.

Each specimen was measured (SL; 1 mm accuracy), weighed (Wt; 1 g) and dissected for internal organs: heart, gonad, liver and spleen, which were weighed. The bodies were cooked, then remeasured and reweighed after dissection of the vertebral column and entrails (We). Weight and length data were used to estimate standard body‐condition coefficients: relative SL/Wt relationship (LWR) and Clark's condition factor (CC = We/SL^3^; Ricker, 2010), followed by anatomical traits, such as the heart‐somatic index (HI), reflecting heart functional capacity, spleen‐somatic index (SSI) as a measure of immunocompetence, the hepato‐somatic index (HSI)—a measure of energy reserves (Šimková et al., 2015)—and vertebrae index (VI), which assesses skeleton ossification. Size of vertebrae was measured along the horizontal axis using digital callipers (accurate to 0.1 mm) of the three sequential vertebrae starting at the plane of the first dorsal pterygiophore. Age‐growth dynamics were estimated according to Fedorčák, Koščo, Halačka, and Manko (2017) on the basis of the total number of annuli on the vertebral body (distances from the vertebra's centre to each annual ring, and to the vertebra edge were measured to the nearest 0.005 mm). Growth dynamics were interpreted as an indication of interclonal competition ability for resources within the shared niche. Fecundity, either absolute (i.e., total number of pre‐matured oocytes in a female) or relative (i.e., absolute fecundity per weight) was estimated as the number of exogenous vitellogenic oocytes in the gonad according to Halačka, Lusková, and Lusk (2000), and Juchno and Boron (2010). Differences in size structure of oocytes among females were estimated according to Halačka et al. (2000) by measuring the diameters of 250 randomly chosen oocytes. We note that these latter measures were performed on a subset of fish of similar size and age to avoid biases due to age differences (Table 2).

To further test for fecundity differences, we allowed four and three hybrid females belonging to ancient (EEN) and recent (ETT) clones, respectively, to mate at will with *C. elongatoides* males under seminatural conditions. We kept each triploid hybrid female in an identical tank, together with a single male, with spawning substratum following the design of Bohlen (1999). After each spawning event, we counted eggs from each clutch and let them develop under standardized conditions until they hatched and reached the third fry stage, when we evaluated their survival rate.

### 2.3 Statistical analyses of data

We used R packages nlme (Pinheiro, Bates, DebRoy, & Sarkar, 2016) and lme4 (Bates, Maechler, Bolker, & Walker, 2015) to analyse differences among fish types in clutch sizes, survival rates, SNP data, site frequency spectra, amino acid radicality and *d*_N_/*d*_S_ ratios. To address specifics of each data set, we used several linear models including linear mixed effects (LME) models, generalized linear models (GLM) and generalized linear mixed‐effects (GLME) models. We obtained *p*‐values for the effect in question by likelihood‐ratio tests (LRT) against the null model. Details about these statistical methods are provided in Supplementary online materials.

## 3 RESULTS

### 3.1 Molecular data

On average, we mapped 17,684,155 reads per sample (all samtools bitwise flags except 0x4) onto a reference *Cobitis* transcriptome (Janko et al., 2018). Within each of the 20,601 contigs, we identified open reading frames (ORF) altogether providing us with 3,945 nuclear ORFs (3,179,571 bp total length) with annotation and sufficient read coverage to identify SNP variants. Mapping the reads onto a published *C. elongatoides* mitogenome (NC_023947.1) sufficiently covered on average 82.1% of the reference, with most reads falling into coding regions (10,902 sites).

#### 3.1.1 Test of relaxed selection on mtDNA

Since we detected some stop codons and heterozygous positions in our putative mtDNA markers, we first attempted to test whether such contigs may not constitute “nuclear mitochondrial DNA” loci (NUMTs). First, we noted that among all samples, stop codons were found in putative mtDNA sequences of only 21 samples (at most a single stop codon per individual has been observed). In addition, some heterozygous positions have been detected in only 31 samples (median = 1.5, max = 48), while 17 samples carried no such positions. Finally, we noted that putative mtDNA contigs were sequenced with above‐average coverage in comparison with other loci (Wilcoxon rank sum test with continuity correction, *p*‐value < 2.2e−16). Such observations seem to contradict the expectations of NUMTs, thus supporting our assumption that these contigs represent mitochondrial DNA.

Smart model selection (SMS), used by the phyml server, selected GTR + G as the best model of sequence evolution (Table S1); the resulting tree is depicted in Figure 2.

Comparison of the two models in relax by likelihood‐ratio test (LRT) did not indicate a preference for the alternative model over the null model (∆AICc = 0.3; *K* = 2.98, *p* = .129, LR = 2.30), which assumed no differences between sexual and asexual branches, and suggested that 86.72% of sites are explained by *d*_N_/*d*_S_ values of 0.02, 7.08% by values of 0.44, and the remaining 6.2% by values of 1.56.

#### 3.1.2 Nuclear DNA

##### SNP categories, relative ages of clones and reliability of SNP calling

**Figure 3:**
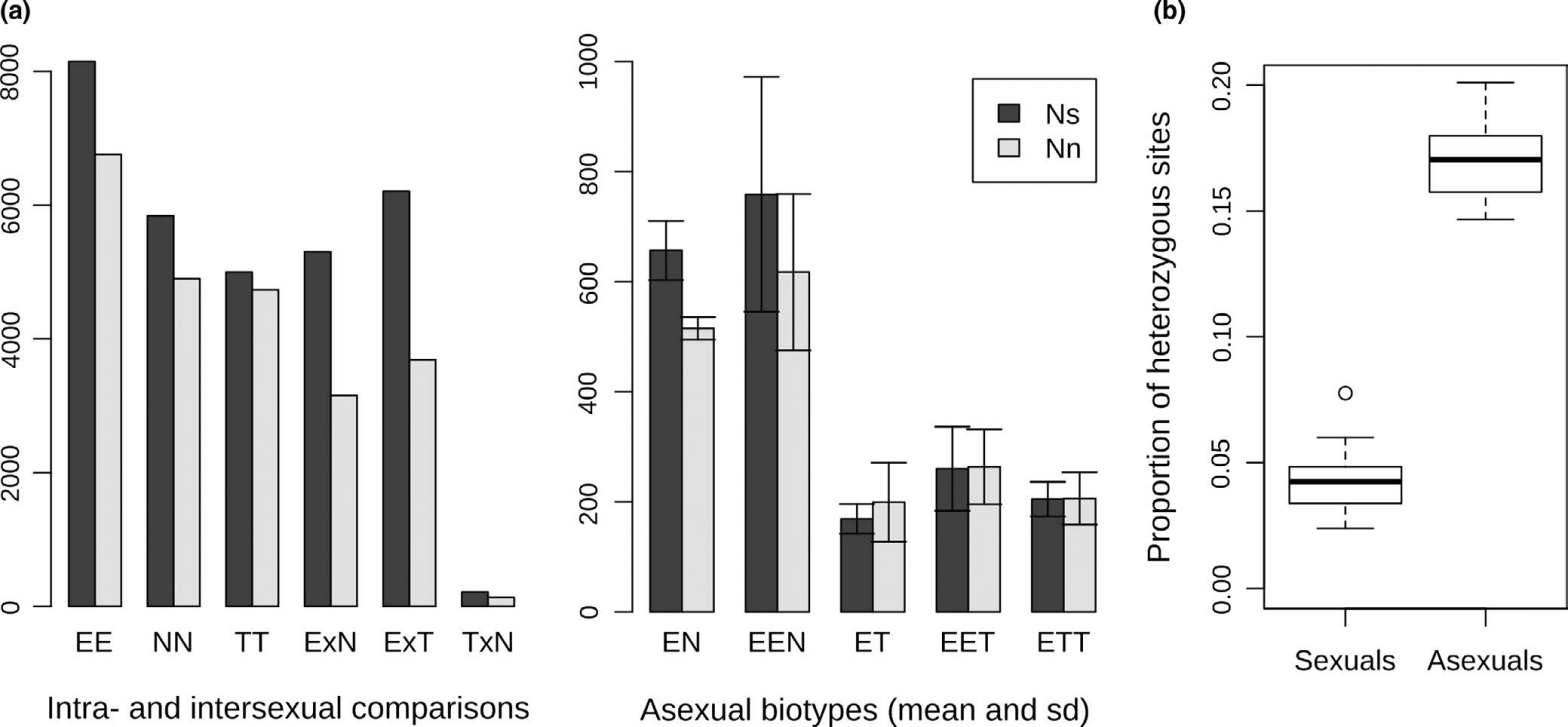
(a) Counts of synonymous (Ns) and nonsynonymous (Nn) mutations. The left panel shows comparisons of polymorphisms segregating within (EE, NN, TT) and fixed between (ExN, ExT, TxN) sexual species. The right panel shows averages (and *SD*) for private asexual SNPs per each hybrid genomotype. Letters correspond to haploid genomes as follows: E = *C. elongatoides*, *N* = *C. tanaitica*, T = *C. taenia*; “x” stands for interspecific comparison, so that, for example ExT indicates comparison performed on *C. elongatoides* and *C. taenia*. (b) Boxplots of observed heterozygosities within sexual and asexual individuals [Colour figure can be viewed at wileyonlinelibrary.com]

Across all nuclear contigs, we discovered 161,139 biallelic SNPs, of which 149,248 were variable within the ingroup. Focusing specifically on annotated ORFs, we categorized 28,878 sites as intraspecific polymorphisms segregating within any of the sexual species. These SNPs were characterized by the prevalence of nonsynonymous substitutions over synonymous ones. We further scored 10,532 fixed interspecific SNP variants, which, in contrast to the intraspecific SNPs, showed the opposite trend, with prevailing synonymous substitutions (Figure 3a). This difference between SNP types was highly significant (GLME with binomial family of error distribution, LRT against null model, *p*‐value = 7.874699e−05). Moreover, the site frequency spectra within each species showed that mutations inducing synonymous changes were significantly more frequent than mutations changing the amino acid (LME, LRT, *p* = 1.61937e−107; Figure S1).

We further noted 8,840 of the so‐called private asexual SNPs. The hypothesis that such sites most likely accumulated after the hybrid origin of clonal lineages is corroborated by the fact that their proportion in hybrids’ genomes was tightly correlated with the mtDNA distance of any clone from its nearest sexual haplotype (Pearson's *r* = 0.94, *p*‐value = 7.135e−14). Specifically, the “hybrid clade I,” that is the oldest asexual lineage, possessed the most divergent mtDNA haplotypes, and the highest proportion of private SNPs (~1.5% of all SNPs). Hybrids from Central Europe with a putative Holocene origin (Janko et al., 2012) possessed haplotypes identical or closely related to those segregating in current sexual populations, and simultaneously had a moderate proportion of private SNPs (~0.2%–0.4%), while the two experimental F1 hybrids possessed the least divergent mtDNA haplotypes, and had a very low proportion of private SNPs, which probably represent false positives due to sequencing errors. The rarity of private mutations in F1s also corroborates the quality of performed SNP calling, which is further strengthened by the fact that our SNP calling recovered 100% matches to sequences of several mitochondrial and nuclear genes previously published by Choleva et al. (2014).

##### Patterns of heterozygosity

**Figure 4:**
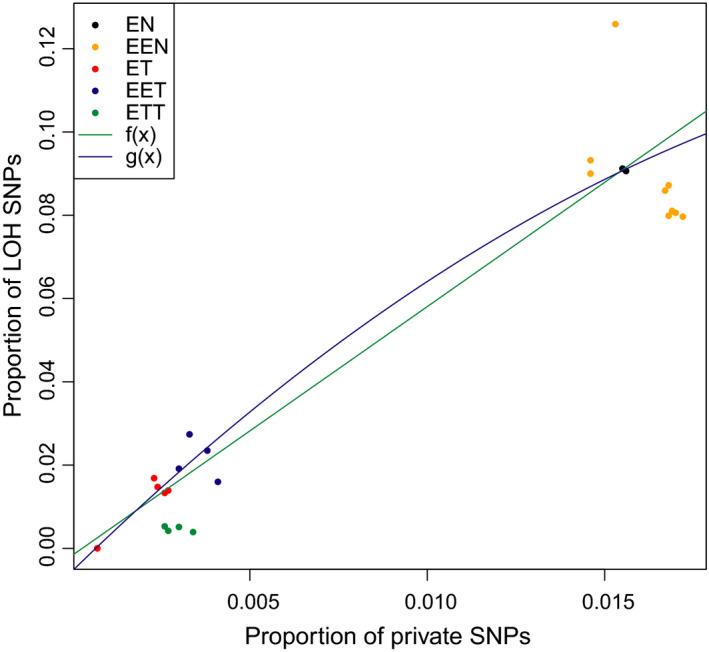
Correlation between accumulation of private mutations and loss of heterozygosity (LOH) events. Green and blue lines represent second‐order polynomial models fitted to diploid data: *g*(*x*) corresponds to the best fit, while *f*(*x*) corresponds to the model with the quadratic coefficient forced to positive values. Note that the second model fits convex parabola, consistent with the assumption that accumulating LOH events erase increasing proportion of private SNPs. Letters correspond to haploid genomes as follows: E = *C. elongatoides*, N = *C. tanaitica*, T = *C. taenia* [Colour figure can be viewed at wileyonlinelibrary.com]

Studied specimens systematically differed in their level of heterozygosity. *C. elongatoides* possessed the highest average heterozygosity of all parental species, and *C. taenia* the lowest (Figure 3b), consistent with its small effective population size (Janko et al., 2018). Asexual individuals had significantly higher genome‐wide heterozygosity than sexuals (Figure 3b; *t* test, *p* = 8.963e−14) with 98.5%–99.8% of “private asexual SNPs” occurring in heterozygous states. The vast majority of fixed interspecific SNPs were in heterozygous states in hybrids’, consistent with expectations. However, every hybrid possessed a small proportion of sites with only one parental allele; that is sites with so‐called loss of heterozygosity (LOH). The proportion of LOH sites in each individual was rather low, and correlated significantly with putative ages of clones, that is either with K2P mtDNA distances from the nearest sexual individual (Pearson's *r* = 0.974, 95% CI = 0.940–0.988, *p*‐value = 3.016e−16) or with proportion of private asexual mutations (Pearson's *r* = 0.955, 95% CI = 0.902–0.980, *p*‐value = 3.286e−14). Hence, the proportion of LOH sites was near zero in experimental F1 hybrids, and highest (~10%) in *elongatoides–tanaitica* (Figure 4).

##### Mutation accumulation and radicality in clones and polyploids

Private asexual SNPs had a significantly higher probability of being nonsynonymous than SNPs fixed among parental species (GLME with binomial family of error distribution, LRT, *p* = .0008). Both intraspecific sexual polymorphisms and private asexual SNPs had similar proportions of nonsynonymous SNPs (binomial GLME, LRT, *p* = .369; Figure 3a). Nevertheless, we noticed that old clones had a significantly lower proportion of nonsynonymous SNPs than young clones (binomial GLME, LRT, *p* = 0.00055) or than sexual species when measured on intraspecific sexual polymorphisms (binomial GLME, LRT, *p* = .12). Altogether, these data indicate no tendency towards a higher nonsynonymous mutation rate in clones, and even suggests the possibility of lower rates in the old clonal lineage.

To evaluate the impact of polyploidization on mutation accumulation, we compared diploid and triploid asexuals within both major types of hybrids (i.e., ET vs. EET and ETT in elongatoides–taenia and EN vs. EEN in elongatoides–tanaitica hybrid types). We noticed that triploids possessed significantly higher proportions of private asexual SNPs compared with diploids (Figure 3a; ET vs. EET & ETT: *t* test, *p* = .006; EN vs. EEN: *t* test, *p* = .001). We also noticed that variance in proportions of private asexual SNPs was generally higher among triploids than among diploids, but these differences were not significant due to small sample sizes (ET vs. EET & ETT: *F* test, *p* = .06; EN vs. EEN: *F* test, *p* = .4). To compare rates of nonsynonymous mutation accumulation between di‐ and triploids, we applied the binomial GLME separately to elongatoides–taenia and elongatoides–tanaitica hybrids, treating individuals within groups as random factors. Private asexual SNPs in diploid ET hybrids accumulated a significantly higher proportion of nonsynonymous mutations compared with EET or ETT triploids (binomial GLME, LRT, *p* = .047), but the differences between EN diploids and EEN triploids were not significant (binomial GLME, LRT, *p* = .42).

When testing the accumulation of large‐effect mutations, we found as expected that interspecific SNPs are significantly less likely to carry premature stop codon mutations than both intraspecific and private asexual SNPs (contingency table, *p* < 1e−15). Asexuals, when considered as one group, had significantly higher incidence of premature stop codons than intraspecific sexual data sets (contingency table, *p* = .016). In our analyses of old and recent clones, we found that the incidence of stop codons did not differ between intraspecific sexual SNPs and private asexual SNPs in old clones (contingency table, *p* = .99), but their incidence was significantly elevated in young clones (contingency table, *p* = 2.4e−6).

**Figure 5:**
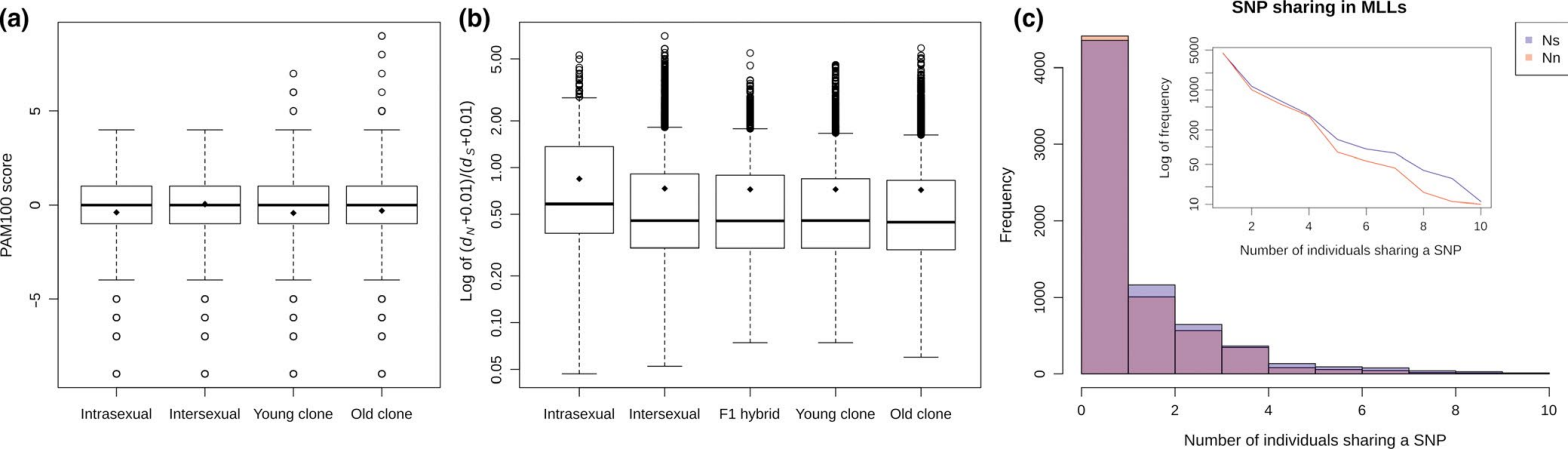
Radicality of amino acid substitutions and *d*_N_/*d*_S_ ratios. (a) Boxplots of PAM100 score distributions of intraspecific, interspecific and private asexual SNPs (the latter split for ancient and recent clones). Lower score means more radical change. (b) Boxplots of *d*_N_/*d*_S_ ratio within sexual species, interspecific pairs, artificial F1 hybrids and hybrid genomotypes split into ancient and recent clones. The horizontal bars indicate the distribution medians, while diamonds indicate the means. (c) Histograms on linear and logarithmic (insert) scales of absolute frequencies of private asexual SNPs within identified clonal lineages. *X*‐axis indicates the number of specimens of the same clone that share given SNP [Colour figure can be viewed at wileyonlinelibrary.com]

We also scored the “radicality” of nonsynonymous mutations using the PAM100 substitution matrix, and compared score distributions among sexual and asexual data sets (Figure 5a). Again, fixed interspecific nonsynonymous substitutions were significantly less radical than intraspecific segregating mutations (LME, LRT, *p* = .0009) or private asexual SNPs (LME, LRT, *p* = .004). However, the radicality of mutations segregating within sexual species (intraspecific polymorphisms) did not differ from that of nonsynonymous private asexual SNPs (LME, LRT, *p* = .339). We observed no significant differences between old and young clones (LME, LRT, *p* = .084). Analogous results were obtained with the BLOSUM90 matrix (data not shown).

##### No evidence for reduced efficacy of selection from *d*_N_/*d*_S_ ratios and mutation frequency spectra

A more formal test of selection was based on the per‐gene distributions of *d*_N_/*d*_S_ ratios in all genomotypes (Figure 5b). A subset of 1,993 ORFs passed a threshold of 50 or more codons fully resolved in all investigated individuals. To address the hybrid origin of asexuals’ variability, we compared the *d*_N_/*d*_S_ ratio of asexuals with that estimated from *elongatoides–taenia* or *elongatoides–tanaitica* sequence pairs, as well as estimates from laboratory F1 hybrid offspring. The usefulness of this approach was corroborated by the fact that *elongatoides–taenia* pairwise *d*_N_/*d*_S_ values did not differ from those in F1‐generation ET hybrids (LME, LRT, *p* = .43).

Most genes in all comparisons had values below 1, consistent with the influence of negative selection. Within‐species pairwise comparisons yielded significantly higher values than interspecific comparisons, and values observed within asexual genomotypes (LME, LRT, *p* < 1e−8). However, the *d*_N_/*d*_S_ ratios calculated from interspecific pairs of sequences were significantly higher than values from old clones (LME, LRT, *p* =0.01), young clones (LME, LRT, *p* = 0.02) or asexuals in general (LME, LRT, *p* = 0.004), which was at odds with predictions based on mutation accumulation hypotheses. Moreover, we found no differences between old and young clones (LME, LRT, *p* = 0.35).

Looking at the distribution of private asexual SNPs, we found that nonsynonymous substitutions often appeared as singletons, while synonymous substitutions were significantly more likely to be shared by two or more members of the same clone (Figure 5c; binomial GLME, LRT, *p* = 5.7e−6). Since the ancient clonal group (hybrid clade I) had the highest sample size, we also looked at its mutation frequency spectra and noticed that nonsynonymous substitutions are again significantly less frequent than synonymous ones (Poisson GLM, LRT, *p* = 1.1e−6).

### 3.2 No evidence for fitness deterioration in old clones

**Figure 6:**
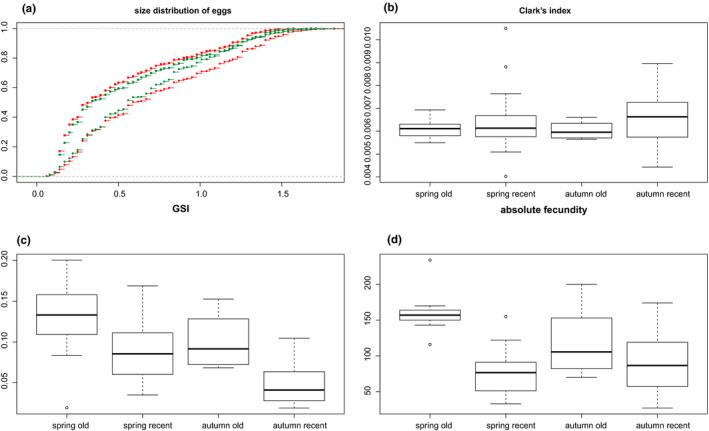
Comparison of selected fitness‐related traits between seasons and ancient versus recent clones. (a) shows the oocyte‐size distributions between ancient (red) and recent (green) clones during the spring (squares) and autumn (diamonds) seasons; (b–d) depict Clark's condition factor, gonadosomatic index (GSI) and absolute fecundities [Colour figure can be viewed at wileyonlinelibrary.com]

We found a significant correlation between fish length and weight (ANCOVA, *F*_1, 56_ = 115.13, *p* = 3.346e−15) and also a significant effect of season, with spring samples having higher SSI (*t*_95_ = 8.34, *p* «0 .000), HSI (*t*_97_ = 3.75, *p* = 0.000) and GSI (*t*_97_ = 4.740, *p* « 0.000) values, and gonads with higher counts of generally smaller oocytes compared with autumn samples (Figure 6a). Some effect of location was also present as fishes from the Złotnica River site had smaller relative SL/Wt ratios (*F*_1, 56_ = 33.56, *p* = 3.291e−07) and slower growth rates (*F*_1, 251_ = 103.3101, *p* < 2.2e−16) than those from the remaining sites.

Taking the aforementioned effects into account, we found no differences between old and young clones in condition‐related measures: LWR (ANCOVA, *F*_1, 56_ = .0018, *p* = .9667), CC (*t*_98_ = −.838, *p* = .404; Figure 6b), HI (*t*_90_ = 0.265, *p* = .791), HSI (*t*_98_ = 1.08, *p* = .282), VI (*t*_58_ = 1.713, *p* = .92) or growth rates (*F*_1, 251_ = 2.11, *p* = .147), except for a significantly higher SSI in old clones (*t*_95_ = −2.05, *p* = .043). Looking at traits related to fecundity, the old clonal lineage possessed significantly higher GSI values than the younger clonal lineages (in spring: *t*_17_ = 2.617, *p* = .018, in fall: *t*_78_ = 5.968, *p* < .000). Egg counts followed this pattern, showing statistical significance in spring samples: absolute fecundity: *t*_17_ = 3.069, *p* = .007; relative fecundity: *t*_17_ = 2.723, *p* = .014 (Figure 6c,d). We also noted differences between lineages in egg size structure. The old clonal lineage possessed larger counts of smaller eggs than the younger clones in the spring period, while the opposite trend was observed in the autumn (permutation Kolmogorov–Smirnov test, *p* « .0001 in both seasons; Figure 6a).

A reproductive experiment corroborated the aforementioned differences, since females of the oldest clone had significantly larger clutches compared with those from the younger clones (average of 394 and 176 eggs per clutch, respectively; GLME with female ID as a random factor, *Z* = 4.364, *p* = 1.28e−05). We noted generally low survival rates (on average ~16% of eggs per clutch hatched and survived till third fry stage), consistent with previous experiments (Bohlen pers.comm.; Janko, Bohlen, et al., 2007; Juchno et al., 2014), but we found no significant differences between old and recent clones in survival (zero‐inflated GLME, *Z* = −.001, *p* = .999).

## 4 DISCUSSION

Various mechanisms have been proposed to explain why sexual reproduction evolved and became a dominant force in nature, but no clear answer has been obtained. We tested whether the mutational deterioration of clones, one of the most cited candidate processes, may serve as an efficient short‐term mechanism explaining the stable coexistence of sexual species with clonal lineages, some reaching ages of up to 300 ky.

### 4.1 No evidence for accumulation of deleterious mutations and fitness decay in asexuals

The observed negative selection prevailed among >3,000 studied nuclear and mitochondrial loci, as indicated by higher proportions of nonsynonymous and more radical mutations, by higher *d*_N_/*d*_S_ values observed within the parental species rather than between the parental species, as well as by generally lower intrapopulation frequencies of nonsynonymous mutations compared with the synonymous ones. This is in line with the hypothesis that nonsynonymous or radical intraspecies polymorphisms may be often mildly deleterious, and may be consequently removed by negative selection before reaching fixation.

We further found that clonal genomes accumulated novel mutations in a time‐dependent manner, so that the older clonal lineages had higher incidence of private asexual SNPs compared with the younger clones or the experimental F1 hybrids, consistent with mutation accumulation models. Nevertheless, the way in which new mutations accumulated contrasts with expectations of a reduced efficacy of selection in asexuals; namely, despite hundreds of thousands of years of clonality in some lineages, any potential reduction in selection efficacy was too weak to provide detectable traces in the asexuals’ mitochondria, and there was no evidence for increased proportions of nonsynonymous or radical mutations among private asexual SNPs in the ancient clone. We also compared *d*_N_/*d*_S_ ratios of real asexuals with values of hybrids simulated from the sequences of the sexual individuals. This approach, which accounts for the effect of fixed interspecific polymorphisms, showed lower‐than‐expected *d*_N_/*d*_S_ values in natural clones, with no difference between the old and the recent clones, again contrasting with predictions of mutation accumulation hypotheses.

The fitness‐related data are also at odds with the prediction of clonal decay since the ancient “hybrid clade I” outperformed the young clones in some important traits, such as GSI and fecundity. Given that proportions of asexuals in mixed sexual–asexual populations often correlate with their competitive success (Hellriegel & Reyer, 2000; Vrijenhoek & Parker, 2009), the evolutionary success of the oldest *Cobitis* clonal lineage is further indicated by its occupation of the largest distribution range of all *Cobitis* clones (Janko, Culling, et al., 2005) and its ability to achieve higher densities than co‐occurring younger clones at many sites (Janko et al., 2012). We have to note that old and recent clones used in our study have different genomic compositions (*elongatoides–tanaitica* vs. *elongatoides–taenia* genome combinations, respectively), suggesting their dissimilarity may not necessarily reflect asexuality‐specific processes but also reflect differences between parental species that were inherited in clonal hybrids. Alternatively, it may be also explained by a longer period of interclonal selection acting upon the older clones than the younger clones (Wetherington, Kotora, & Vrijenhoek, 1987; Wetherington, Weeks, Kotora, & Vrijenhoek, 1989). Nevertheless, the absence of a detectable effect on fitness continues to indicate that even the oldest *Cobitis* clones successfully coexist and compete for resources with recent clones and sexual species.

### 4.2 How do asexuals escape mutational deterioration?

Altogether, the acquired data provide no evidence for reduced efficacy of the selection in asexuals, suggesting that mutation accumulation may not notably hamper the demographic and the competitive performances of the *Cobitis* clones even after 300 ky of asexuality. What are the reasons behind these observations?

First, the observed patterns were unlikely to be methodological artefacts, since our study covered most of the *Cobitis* distribution range and provided a relatively high number of SNPs that were validated in the experimental F1 hybrids and previously published sequences, and estimated values of the phenotypic traits did not deviate from previous reports of the *Cobitis* genomotypes, including the polyploids (Halačka et al., 2000; Juchno & Boron, 2010). On the other hand, we note that the inherent biological nature of the studied organisms may have affected our data since comparisons between asexuals and their sexual counterparts (or simulated hybrids) conflate the variability frozen by hybridization in the distant past with that segregating within the contemporary sexual populations. Additionally, comparisons between the old and the recent clones potentially suffer owing to the presence of different species in their ancestries. Ideal asexual systems to study such processes would include both young and old clonal lineages, as well as sexual individuals with the same genomic compositions. However, even in the case of nonhybrids, natural asexuals often differ from sexuals in other ways than just the mode of reproduction. For instance, asexuality is often connected with polyploidy (Lundmark, 2006), which may affect many biological processes to a greater extent than hybrid origin itself (e.g., Maciak et al., 2011).

Carefully addressing these pitfalls, we clearly documented that mutations did accumulate in time, as predicted by the clonal decay model, but ~300 ky of asexuality did not leave detectable traces of the deleterious mutation accumulation or decreased clonal fitness. This study adds to the growing list of publications that have failed to detect traces of clonal decay in asexual organisms, including hybrids (e.g., Pellino et al., 2013; Warren et al., 2018).

What does the growing amount of negative evidence tell us about the validity of mutation accumulation theories for evolution of sex and clonality? Commonly, negative results have been attributed to special mechanisms enabling investigated asexuals to avoid mutation accumulation, such as deviations from a strict clonality including minimal sex, ameiotic recombination, gene conversions, beneficial effects of polyploidization or an increased efficiency of DNA repair (e.g., Maciver, 2016; Roach & Heitman, 2014; Schön & Martens, 2003; Warren et al., 2018). However, given the available data, it is unlikely that any such mechanism may have efficiently slowed down the clicks of the ratchet in the *Cobitis*.

Refuting cryptic sexuality in predominantly clonal organisms is notoriously difficult (e.g., Birky, 2010), but available evidence argues against its role in the *Cobitis*. Extensive crossing experiments have never documented allelic segregation (Choleva et al., 2012; Janko, Bohlen, et al., 2007; Janko et al., 2018), and Majtánová et al. (2016) reported remarkable stability of the hybrids’ karyotypes with no large‐scale chromosomal restructuring or recombination. The current study also shows that the vast majority of the private hybrid SNPs occur in heterozygous states (between 98.5% and 99.8%), which corroborates the hypothesized clonal reproduction, with new mutations occurring on one chromosome with little possibility of recombination between the homologues or coalescence between the alleles. Nevertheless, the existence of LOH at fixed interspecific positions indicates that asexual organisms’ alleles may occasionally recombine, convert or vanish due to hemizygous deletions. However, three lines of evidence argue against a major impact of LOH on the speed of ratchet clicks in the *Cobitis*.

First, even the oldest *Cobitis* clones accumulated LOH events in only ~10% of the studied genes over ~300 ky, which strikingly contrasts with other asexual organisms, where such processes have been hypothesized to interfere with the accumulation of deleterious mutations. For example, Tucker, Ackerman, Eads, Xu, and Lynch (2013) and Sunnucks, England, Taylor, and Hales (1996) showed orders of magnitude of higher rates of conversions and deletions of individual genes, and even entire chromosomal arms. Second, the efficiency of LOH events in erasing potentially deleterious mutations is expected to increase with the clonal age. This is because, rare LOH events may likely happen on different genes from new mutations in the recent clones, while in the older clones any emerging LOH event has a higher chance of occurring in the genes with existing mutations. Therefore, if LOH events were to attenuate the ratchet by overwriting deleterious mutations, we would expect an exponential correlation between the proportions of LOH and the private asexual SNPs because LOH would erase a disproportionately higher proportion of acquired mutations in old clones compared with the younger ones. As evidenced in Figure 4, our data offer no support for such a deviation from linearity (note that we focused on diploid clones here since the presence of three homologues in triploids complicates the detection of LOH). Finally, only <1.5% of the private SNPs were in homozygous state, probably resulting from LOH events. If we assume that LOH events are more or less symmetrical with respect to preserving or losing the new or ancestral allele, it suggests that only ~3% of the private asexual mutations could have been converted. Of course, if new mutations tend to be deleterious, the selection might have preferred one direction of LOH, but altogether, our data indicate that genome restructuring likely has no major effect on the clicks of the ratchet.

Polyploidization is another mechanism that may refresh a clonal genome by temporarily masking deleterious mutations, although it also frees gene copies to mutate, ultimately increasing the per‐genome mutation rate (Otto & Whitton, 2000). Consistent with this expectation, we found higher numbers of private asexual SNPs in triploid loaches than in diploids, but the differences were relatively small. This may suggest that the contemporary triploids may have spent considerable parts of their history as diploid clones before polyploidization, leaving relatively little time for accumulation of extra mutations. Importantly, we found no evidence for the increased nonsynonymous mutation rates in triploids. Hence, although polyploidization likely plays a very important role in clonal evolution, at least in the *Cobitis*, its major benefits do not seem to stem from a deleterious mutation masking; after all, the diploid clones have persisted at the Balkan together with their triploid derivatives at least since the last interglacial period (Janko, Culling, et al., 2005). Major benefits of triploidy may instead relate to other effects, such as metabolic changes invoked by the modified cell architecture, the altered dosage of parental genomes or the modified crosstalk between the allopolyploids’ genomes due to different stoichiometric relations between transcription factors and their binding sites (Bartoš et al., 2019; Beukeboom & Vrijenhoek, 1998; Maciak et al., 2011).

### 4.3 The null model of clone persistence assumes delayed effects of accumulated mutations

In summary, although we may not rule out the existence of some mechanisms counteracting the effects of clonality, the aforementioned candidate mechanisms do not appear strong enough to stop or considerably slow the ratchet in the *Cobitis*. Instead, we propose that the apparent absence of signs of clonal decay, as in the *Cobitis* and other similar cases, may have a much simpler reason, which we propose as a null hypothesis for any tests of clonal decay; namely, the simplest plausible explanation is that the reduction in efficacy of a purifying selection caused by asexuality is not strong enough to allow sufficient mutation accumulation that will compromise the performance of clones over several hundreds of thousands of generations.

Consistent with this explanation, we found similar frequency spectra of non‐/synonymous mutations in the sexual and the asexual populations, indicating that selection efficiently restricts the spread of putatively deleterious mutations even in the clones. The counterintuitive observations of lower nonsynonymous mutation loads and the incidence of premature stop codons in old clones may thus be explained by the classical population genetic theory, since a selection‐based removal of the deleterious mutations requires varying amounts of time depending on the selection coefficient, the population size and the genetic background. It follows that recent clones, despite having acquired lower absolute numbers of mutations, will possess a higher fraction of nonsynonymous private asexual mutations, or more radical SNPs.

Different mutation loads in the old and the young clones may thus reflect a time‐lag necessary to remove the slightly deleterious mutations (Johnson & Howard, 2007) rather than some special mechanisms that alleviate the mutation accumulation. Paradoxically, the delay in fitness decay may be caused directly by clonal reproduction because the maintenance of heterozygosity may mask the negative effects of acquired deleterious recessives (Halligan & Keightley, 2003) until new mutations accumulate in high numbers (Otto, 2007) or become exposed to selection in the homozygous states (Guex et al., 2002; Leslie & Vrijenhoek, 1978, Leslie & Vrijenhoek, 1980). This was in line with recent reanalysis of published sequences of asexual animals (Janko et al., 2011), which showed that significant deviations from neutrality, indicative of selection against ageing clones, could only be detected in asexual complexes when clones achieved substantial ages beyond 1 million generations.

Hence, even if a clonal lineage were to accumulate deleterious mutations since its birth, it may take considerable time before they start to negatively affect the evolutionary lifespan of that clone. In younger asexual complexes, the replacement of clonal lineages may be dominated by a more neutral drift‐like process of the clonal turnover, which does not depend on the effects of accumulated mutations (Janko et al., 2008). By analogy to a genetic drift, the clonal turnover predicts that a major portion of clonal diversity may be formed by the young clonal lineages, which did not have enough time to spread, while ancient lineages will be less diverse but more geographically widespread. Interestingly, both predictions have been met in the *Cobitis* and in some other taxa (Janko, 2014; Janko et al., 2012; Quattro, Avise, & Vrijenhoek, 1992).

## 5 CONCLUSIONS

To date, the existence of successful clones in nature has usually been explained by their special abilities to avoid accumulation of the deleterious mutations. Our study shows that even if clones clearly accumulate mutations as they age, the reduction in efficacy of selection due to asexuality may not be sufficient to cause their fitness to deteriorate, even after hundreds of thousands of generations of asexuality. Thus, although mutation accumulation may ultimately drive any clone to extinction, individual clones may be replaced by more recent clones in a drift‐like process of the clonal turnover if the influx rates of new clones are high (Janko et al., 2008). Clones in such complexes may simply not be given an opportunity to become old enough for accumulated mutations to start to manifest their effects. Successful and widespread clones may therefore often represent merely temporary winners in the ongoing turnover race.

This may seem trivial, but it has serious implications for our understanding of the persistence of sex: Mutation accumulation may be relevant to the survival of clones after they reach some substantial age, but before they do so, the persistence of sexual species in mixed sexual–asexual complexes must rely on different mechanisms that offer short‐term advantages to sex.

## AUTHOR CONTRIBUTIONS

K.J. designed the experiments. K.H., J.F., J.Koš. and NI performed the experiments. J.Koč., J.R., J.P., J.Kot. and K.J. analysed data. J.Koč., J.Kot., J.R. and K.J. contributed to writing the manuscript.

## Supporting information


**Figure S1**

**Table S1**

**Data S1**

**Data S2**

**Data S3**

**Data S4**

**Supplementary Material**

**Supplementary Material**

## Data Availability

Raw sequencing data are available from the NCBI SRA with accession PRJNA639562. Input files are provided as supplementary files.
