# Supplementary material for: No evidence for accumulation of deleterious mutations and fitness degradation in clonal fish hybrids: Abandoning sex without regrets
Source: Mol Ecol. 2020 Aug 4;29(16):3038–55. doi: 10.1111/mec.15539 (PMC7540418; doi:10.1111/mec.15539)
Supplement: Supplementary file 1 — Figure S1 [file MEC-29-3038-s001.pdf]

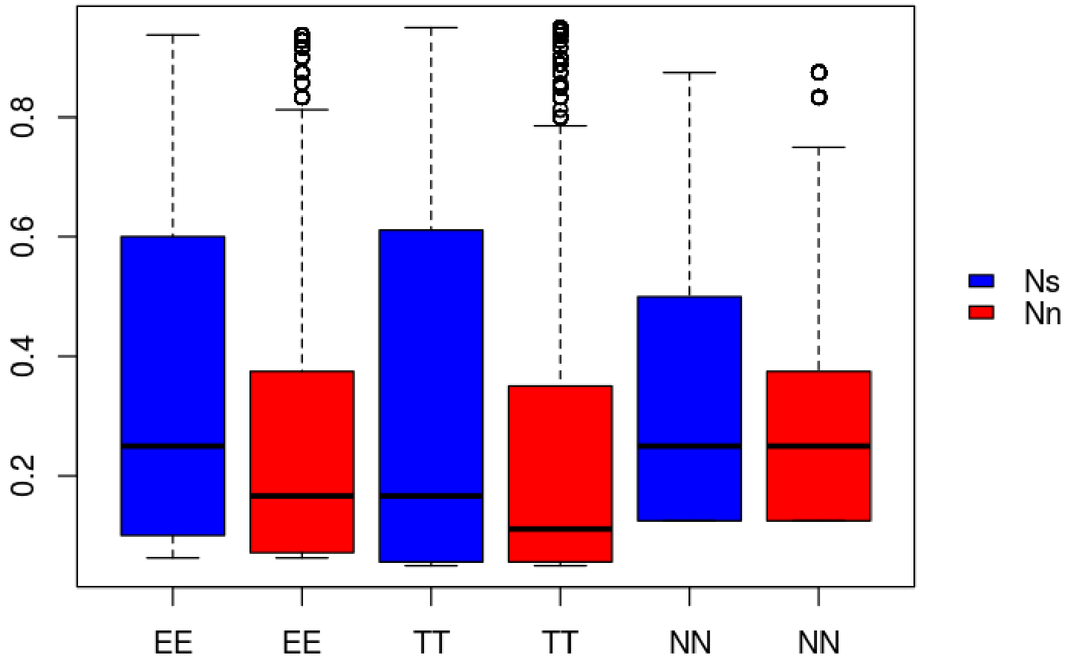

Supplement 2: Site frequency spectra of segregating sites within sexual species. Boxplots demonstrate the distribution of frequencies of segregating derived alleles in each species. As derived alleles, we define those which differ from the sister species and hence are putatively mutated in the target species. Species are indicated by letters: EE (*C. elongatoides*), NN (*C. tanaitica*), TT (*C. taenia*). The frequencies of nonsynonymous alleles were significantly lower than of synonymous ones as tested by linear mixed-effect model with species considered as a random factor (LME LRT against null model *p*-value = 1.025496e-111).
