# Supplementary material for: No evidence for accumulation of deleterious mutations and fitness degradation in clonal fish hybrids: Abandoning sex without regrets
Source: Mol Ecol. 2020 Aug 4;29(16):3038–55. doi: 10.1111/mec.15539 (PMC7540418; doi:10.1111/mec.15539)
Supplement: Supplementary file 7 — Supplementary Material [file MEC-29-3038-s007.docx]

No evidence for accumulation of deleterious mutations and fitness degradation in clonal fish hybrids: Abandoning sex without regrets

Supplementary methods

Jan Kočí, Jan Röslein, Jan Pačes, Jan Kotusz, Karel Halačka, Ján Koščo, Jakub Fedorčák, Nataliia Iakovenko, Karel Janko

# Statistical models

For site frequency spectra we performed LME analysis of relationship between allele frequency (response) and synonymity of substitutions (fixed effect), with species accounted as random effect. For radicality of amino acid substitutions and *d*_N_/*d*_S_ ratio we used LME to assess differences between all categories of interest (treated as fixed effect; see Tab. 1). PAM100 score or *d*_N_/*d*_S_ ratio were used as response and particular species or hybrid genomotypes within categories as random effect. To test whether synonymous mutations are more prevalent than nonsynonymous ones, we applied GLME with binomial family of error distribution, where we treated synonymy of mutations as binary response, category as fixed effect and species or genomotypes within categories as random effect. We then used GLME with binomial family of error distribution to test if sharing or not sharing a particular SNP among two or more samples from the same clonal lineage (a binary response) could be explained by synonymity of substitution (fixed effect), with clone ID taken as random factor. We also tested sharing of SNPs within the most populous clonal lineage (Clone ID 9; see Tab. 1) using GLM with Poisson family of error distribution.

We determined length – weight relationship (LWR) using the equation of Le Cren (1951): Wt = *a*SL*^b^*; transformed by taking the natural logarithm into ln (Wt) = ln (*a*) + *b* ln (SL); where *a* is the regression intercepts, and *b* is the slope of the regression coefficient. For SL-Wt evaluation we fitted a linear model (LM) with ln (Wt) as the response variable, and ln (SL), clonal affiliation, sample locality and their two-way interactions as explanatory variables. We estimated significance of each regression by ANOVA and compared regressions with ANCOVA.

We estimated growth with the use of FSA package according to Ogle (2013): back-calculated lengths for subsequent year of life of each individual were calculated using the direct proportion method (Dahl, 1907; Lea, 1910) as the regression between SL and R: BCL = SL(rn· R^–1^); where BCL is back calculated length at the annulus, SL is length at capture, rn is age structure radius at the annulus, and R is age structure radius at capture. In order to determine whether back-calculated length-at-age differed for the old vs. young clones, we fitted a second-degree polynomial (quadratic) linear model. The model’s right-side contained explanatory variables 1) the group factor variable (type of clone), with 2) increments (inc) and 3) the squared version of increments (incSqrd) and also 4) the sample locality as quantitative explanatory variables (quadratic regression). The model also included the two-way interaction between above mentioned quantitative explanatory variables. We submitted the model to ANCOVA in the order to extract the Type I and Type III sum of squares according to Fox and Weisberg (2010). Details of methods are described in Fedorčák et al. (2017).

We tested between-group comparisons of other condition- and fecundity-related features on ln-transformed data using two tailed t-test assuming equal variance, preceded by testing for normality with the Shapiro-Wilk’s and Levene’s test for homogeneity of variance.

We compared the structure of oocyte-size in gonads among genomotype-season groups with the permutational Kolmogorov-Smirnov test (KS) by comparing the KS value for differences of two empirical cumulative density curves with the null distribution of the KS values, which we estimated based on 1,000 comparisons between pseudoreplicates of data resampled without replacement from pooled dataset. We tested the differences in clutch sizes between old and recent clonal triploids with the GLME with Poisson family of error distribution and treating females identity as a random effect. Due to large amount of zeros, we tested the differences in survival rate in R with the help glmmTMB library (Magnusson et al., 2017) using the zero inflated GLME with binomial error distribution and treating female identity as a random effect.

# References

Dahl K. (1907). The scales of herring as a means of determining age, growth and migration. *Report of the Norwegian Fisheries and Marine Investigations* 2, 1–39.

Fedorčák, J., Koščo, J., Halačka, K., & Manko, P. (2017). Growth differences in different biotypes of the hybrid complex of *Cobitis elongatoides* × *Cobitis tanaitica* (Actinopterygii: Cypriniformes: Cobitidae) in the Okna River (Danube River basin), Slovakia. *Acta Ichthyologica Et Piscatoria*, 47, 125–132. doi:10.3750/AIEP/02059

Fox, J., & Weisberg, H. S. (2010). An R Companion to Applied Regression (2 edition). Thousand Oaks, Calif: SAGE Publications, Inc.

Lea E. (1910). On the methods used in the herring-investigations. *Conseil Permanent International pour l'Exploration de la Mer* 53, 7-174.

Magnusson, A., Skaug, H. J., Nielsen, A., Berg, C. W., Kristensen, K., Maechler, M., ... & Brooks, M. E. (2017). glmmTMB: Generalized linear mixed models using a template model builder. R package version 0.1.3.

Ogle D. H. (2013). FSA: fisheries stock analysis. R package version 0.3.5. R Foundation for Statistical Computing, Vienna.
