# Supplementary material for: No evidence for accumulation of deleterious mutations and fitness degradation in clonal fish hybrids: Abandoning sex without regrets
Source: Mol Ecol. 2020 Aug 4;29(16):3038–55. doi: 10.1111/mec.15539 (PMC7540418; doi:10.1111/mec.15539)
Supplement: Supplementary file 8 — Supplementary Material [file MEC-29-3038-s008.docx]

No evidence for accumulation of deleterious mutations and fitness degradation in clonal fish hybrids: Abandoning sex without regrets

Supplementary data

Supplementary genetic data are provided in TSV format (Tab-separated values). Variant positions are expressed using IUPAC notation. Supplementary phenotypic data are provided in XLSX format.

**Supplementary data 1 – Table of variants for sexual species:**

- locus
- position
- biotype = either pure species (EE, NN, TT) or interspecific polymorphisms (E-N, E-T, T-N)
- Ns = synonymous substitution
- Nn = nonsynonymous substitution
- ances = frequency of ancestral variant
- var1 = frequency of variant 1
- var2 = frequency of variant 2
- aa1 = amino acid encoded by variant 1
- aa2 = amino acid encoded by variant 2

**Supplementary data 2 – Table of variants for asexual hybrids:**

- locus
- position
- sample = sample ID
- biotype = asexual hybrids (EEN, EET, EN, ET, ETT)
- Ns = synonymous substitution
- Nn = nonsynonymous substitution
- seq.s = variant from parental (sexual) species
- seq.a1 = asexual variant 1
- seq.a2 = asexual variant 2
- aa.s = amino acid encoded by variant from parental (sexual) species
- aa1 = amino acid encoded by variant 1
- aa2 = amino acid encoded by variant 2

**Supplementary data 3 – Analysis of radicality of amino acid substitutions:**

- locus
- position
- biotype = pure species (EE, NN, TT), interspecific polymorphisms (E-N, E-T, T-N), or hybrids (EEN, EET, EN, ET, ETT)
- aa1 = amino acid encoded by variant 1
- aa2 = amino acid encoded by variant 2
- EBLOSUM90 = score according to BLOSUM90 matrix (from Emboss package)
- EPAM100 = score according to PAM100 matrix (from Emboss package)

**Supplementary data 4 – Phenotypic and fitness data of young and old clones:**

This file contains four datasets for the phenotypic and fitness analyses, each on a separate sheet. For the first three datasets, the samples are organized in rows, while for egg size dataset, the samples are organized in columns.

- Clark’s index
  - clone_category = young / old
  - season = spring / fall
  - clarks_index
- Gonadosomatic index (GSI)
  - clone_category = young / old
  - season = spring / fall
  - gsi
- Relative fecundity
  - clone_category = young / old
  - season = spring / fall
  - relative_fecundity
- Egg size
  - data on egg size from 20 samples and 250 eggs each (see Methods):
    - 5 old spring
    - 5 old fall
    - 5 young spring
    - 5 young fall
